# Maternal precarity and HPA axis functioning shape infant gut microbiota and HPA axis development in humans

**DOI:** 10.1371/journal.pone.0251782

**Published:** 2021-05-20

**Authors:** Johanna R. Jahnke, Jeffrey Roach, M. Andrea Azcarate-Peril, Amanda L. Thompson

**Affiliations:** 1 Department of Anthropology, University of North Carolina at Chapel Hill, Chapel Hill, North Carolina, United States of America; 2 Carolina Population Center, University of North Carolina at Chapel Hill, Chapel Hill, North Carolina, United States of America; 3 Research Computing Center, University of North Carolina, Chapel Hill, Chapel Hill, North Carolina, United States of America; 4 UNC Microbiome Core, Center for Gastrointestinal Biology and Disease, School of Medicine, University of North Carolina, Chapel Hill, North Carolina, United States of America; 5 Department of Medicine, Division of Gastroenterology and Hepatology, School of Medicine, University of North Carolina, Chapel Hill, North Carolina, United States of America; 6 Department of Nutrition, Gillings School of Global Public Health, University of North Carolina at Chapel Hill, Chapel Hill, North Carolina, United States of America; University of Illinois at Urbana-Champaign, UNITED STATES

## Abstract

**Background:**

Early life exposure to adverse environments, and maternal stress in particular, has been shown to increase risk for metabolic diseases and neurobehavioral disorders. While many studies have examined the hypothalamic-pituitary-adrenal axis (HPA axis) as the primary mechanism behind these relationships, emerging research on the brain-gut axis suggests that the microbiome may play a role. In this study, we tested the relationships among maternal precarity and HPA axis dysregulation during the peripartum period, infant gut microbiome composition, and infant HPA axis functioning.

**Methods:**

Data come from 25 mother-infant dyads in the Galápagos, Ecuador. Women completed surveys on precarity measures (food insecurity, low social support, depression, and stress) and gave salivary cortisol samples during and after pregnancy. Infant salivary cortisol and stool were collected in the postpartum. Statistical significance of differences in microbial diversity and relative abundance were assessed with respect to adjusted linear regression models.

**Results:**

Maternal precarity was associated with lower diversity and higher relative abundance of *Enterobacteriaceae* and *Streptococcaceae* and a lower relative abundance of *Bifidobacterium* and *Lachnospiraceae*. These patterns of colonization for *Enterobacteriaceae* and *Bifidobacterium* mirrored those found in infants with HPA axis dysregulation. Maternal HPA axis dysregulation during pregnancy was also associated with a greater relative abundance of *Veillonella*.

**Conclusions:**

Overall, exposures to precarity and HPA axis dysregulation were associated with an increase in groups that include potentially pathogenic bacteria, including *Enterobacteriaceae*, *Streptococcaceae*, and *Veillonella*, and a decrease in potentially protective bacteria, including *Bifidobacteri*um and *Lachnospiraceae*, as well as a decrease in overall diversity.

## Introduction

Important both developmentally and evolutionarily, growth within the first 1000 days, the period from conception through the second year of life, constitutes a sensitive period during which an individual’s phenotype is plastic. Research on the developmental origins of health and disease (DOHaD) has shown that stress experienced *in utero* and in early life shapes long-term risk for metabolic diseases, including obesity, cardiovascular disease, and diabetes [1] as well as neurobehavioral disorders in offspring even when controlling for adverse birth outcomes [2,3].

Nonetheless, the mechanisms by which perinatal stress is embodied within mother-infant dyads are not yet fully understood, and studies have used various measures of maternal precarity (stress, depression, socioeconomic status, etc.) to assess this question. Many animal models have primarily linked prenatal stress exposure to hypothalamic-pituitary-adrenal axis (HPA axis) dysregulation [4], but studies with humans have not consistently identified a mechanism for the relationship, and research has not fully explored other pathways for these changes in development, in particular, the role of the gut microbiome. Further, unfavorable shifts in the infant gut microbiome, termed dysbiosis, have been associated with the same long-term disease risks as HPA axis dysregulation, namely increased risk for metabolic [5] and neurobehavioral disorders [3], suggesting that the gut microbiome could be a candidate for involvement in this pathway.

Most microbial species develop a symbiotic relationship with their host that promotes healthy development, educates the immune system, supports the development of gut function, regulates intestinal barrier function, protects against infection, promotes food tolerance, and supports central nervous function and the neuroendocrine system including the HPA axis [5–7]. Generally, the healthy gut maintains a state of homeostasis, in which it balances microbial communities, epithelial tissue of the intestine, and the immune system [8]. However, environmental disturbances, including changes in the immune system, diet, stress, and exposures to xenobiotics (antibiotics and anti-cancer medications), among other exposures, can induce dysbiosis [8], which has been associated with risk for obesity, metabolic disease, autoimmune disease and allergy, and intestinal inflammation [5,9].

Further, recent research has shown that the microbiota communicate bidirectionally with the central nervous system (CNS) [10] and thus the gut microbiota may both influence and be influenced by brain function [3,7]. The HPA axis, in particular, has been at the center of much of this work, and studies have found that differences in HPA axis function are associated with differences in gut microbiome composition [11]. In particular, studies have found that germ-free mice (those with no commensal microbiota) have a higher stress response than pathogen-free mice [12], and that pre-treating rats with probiotics reduces hyper-reactivity of the HPA axis [13]. Other research on this pathway has found that psychological disorders that influence the HPA axis, including anxiety, stress, autism, and depression, are associated with differences in gut microbiome composition [14].

The peripartum period offers a unique opportunity to assess how maternal stress may be embodied in offspring through the microbiome, conferring microbiome dysbiosis with long-term health consequences intergenerationally. During pregnancy, maternal stress has been shown to alter maternal vaginal [15,16] and gut microbiota [17,18], and stress-induced changes to maternal microbiota could be transferred to offspring *in utero*, during parturition, or both. The long-held hypothesis that infants are born sterile [19] has been challenged by recent research that demonstrates that the placenta [20] and meconium [21] contain fragments of bacterial DNA, suggesting that infants may encounter bacterial exposures before birth [22]. Proponents of this hypothesis suggest that maternal microbiota can be transferred to a developing fetus through the bloodstream and placenta [23], enabling shifts in a woman’s microbiome to be passed to the fetus during pregnancy.

While the question of newborn sterility remains open, perturbations in a woman’s microbiota during pregnancy may, nonetheless, be transferred vertically to the infant during parturition, and thus serve as foundational microbial communities [6,15,16]. Research on these pathways has found evidence that maternal stress during pregnancy influences offspring microbiome composition in both animal [15,17] and human models [24].

Infant stress in the postpartum period has also been associated with differences in infant gut microbiome development. Early life stress has been found to influence the composition of bacterial microbiota in rhesus monkeys [25] and have long-term effects on the gut microbiota of rats [2]. Further, postpartum maternal stress may influence infant stress, and thus infant microbiome composition through a variety of pathways. First, postpartum maternal stress can compromise a mother’s mental health and caregiving behaviors [26,27], which have each been found to be associated with HPA axis dysregulation in offspring [28–31]. Second, high postpartum maternal cortisol [32,33] and microbial shifts [34] could be transferred to the infant during breastfeeding [34]. In addition to shaping the infant HPA axis, which communicates with the infant’s microbiome, postpartum maternal stress could shape an infant’s physical environment, which has been shown to influence the gut microbiome [23]. These studies demonstrate how stress during the peripartum period can have significant and long-term effects on the development of the gut microbiome, but few studies have directly addressed these questions in humans.

In the present study, we examine the relationships among measures of maternal precarity during and after pregnancy and the development of the infant gut microbiome in humans in the Galápagos Islands, where food insecurity [35–38] and social isolation [38,39] contribute to the island’s high rates of stress and depression [36,40]. Outside of the context of the Galápagos these measures have also been linked. Food insecurity has been associated with higher stress [41,42] and depression [41,43,44] in various contexts, while social support has been associated with lower stress [45] and depression [46–48]. Due to the importance of these factors in the Galápagos Islands, we have selected food insecurity, low social support, stress, and depression symptoms to serve as measures of precarity.

**Fig 1:**
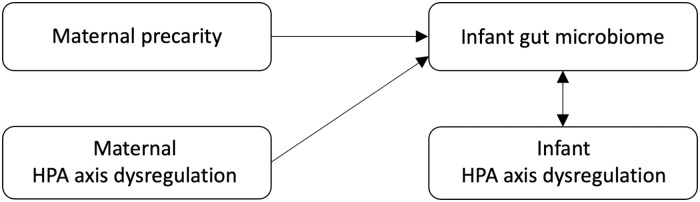
Conceptual model.

In this study in the Galápagos, we ask: 1) Is peripartum maternal precarity, measured through food insecurity, low social support, depression symptoms, and stress associated with differences in infant gut microbiota diversity and predominant taxa? 2) Is peripartum maternal HPA axis dysregulation associated with infant gut microbiota diversity and taxa? And, 3) Are differences in infant gut microbiome composition associated with differences in infant HPA axis regulation? Our conceptual model is shown in Fig 1.

We hypothesize that maternal precarity measures, maternal HPA axis dysregulation, and infant HPA axis dysregulation will be associated with lower abundances of lactobacilli and bifidobacteria in infant stool, as high maternal stress has been associated with these shifts in both human [24] and animal models [16,22,49].

## Materials and methods

### Setting and sample

The data were collected over 12 months from January through December 2018. Participants were recruited from a public hospital on the Galápagos’ San Cristóbal island using purposive sampling (N = 25) [50], through which we selectively recruited pregnant women. The hospital is free for all residents and is the only hospital on the island, allowing the research team to screen all pregnant women in the community within the recruitment period. Inclusion criteria required that women be between the ages of 18 and 50 years old and plan to give birth on the island. Of those contacted who were eligible, only four decided to not take part in the study. Of those contacted who were ineligible, 12 planned to give birth on the mainland, four had already moved off the island, and three had serious pregnancy complications, and thus were excluded. Based on annual birth rates, we estimate that we enrolled more than 50% of the births on the island in 2018. Here we present a complete case analysis for those with microbiome data, which limits our original sample size of 38 participants to 25 participants.

Visits with participants were conducted once during pregnancy and three times in the postpartum period. The prenatal visit was conducted at 34–36 weeks. Postpartum visits were conducted at three days postpartum, one month postpartum, and two months postpartum. For each mother-infant dyad, the research team collected semi-structured interviews; surveys on stress, depression, social support, and food insecurity; maternal and infant saliva samples; and infant stool samples at 2 months of age. All visits were conducted in the participants’ homes, places of work, the hospital, or the Galápagos Science Center (GSC). All participants provided written informed consent prior to participation under appropriate protocols approved by the Institutional Review Boards for the University of North Carolina at Chapel Hill (17–0272) and *Universidad San Francisco de Quito* (2017-163E). This project was also approved by Ecuador’s Ministry of Public Health. The authors have no conflicts of interest to disclose.

### Measures

*Infant stool collection*. Infant stool samples were collected when the infant was two months old. The mean (SD) infant age at stool collection was 59.8 (5.2) days. For infant stool collection, mothers were given detailed oral and written instructions for the collection of the stool sample as well as a sample collection kit, including gloves, a small plastic spoon, a small plastic container. Mothers were asked to collect a small (roughly 400 mg) amount of stool from their infant’s diaper and store it in the sealed plastic container in their own freezer until it could be collected by the research team later that day. Stool samples were then stored frozen at the Galápagos Science Center until they were transported to the Microbiome Core Facility at the University of North Carolina at Chapel Hill (UNC) for analysis.

Various measures of maternal precarity were taken during and after pregnancy. In this analysis, we included measures of food insecurity, stress, depression, and low social support as maternal precarity exposures. Scores on each of the surveys were analyzed categorically for ease of analysis with microbiome data.

#### Food insecurity

Food insecurity was assessed using the Latin American and Caribbean Food Security Scale (ELCSA) [51], with higher scores indicating a higher level of food insecurity. On this scale, a score of 0 indicates a food secure household, scores from 1–5 indicate mild household food insecurity, scores from 6–10 indicate moderate household food insecurity, and scores of 11–15 indicate severe household food insecurity [51]. In our analyses, we grouped food secure and low food insecurity into one category, which will be referred to as “food secure” (88% of sample) and we grouped moderate and high food insecurity into another category that will be referred to as “food insecure” (12% of sample).

#### Stress

The Perceived Stress Scale (PSS) [52] was used to assess maternal chronic stress by measuring the degree to which situations are perceived as unpredictable, uncontrollable, and burdensome [52]. We used a Spanish version of this instrument that has been tested for reliability, validity, and sensitivity in Spanish-speaking contexts [53]. The PSS is scored from 0 to 40, with scores of 0–13 indicating low stress, scores of 14–26 indicating moderate stress, and scores 27–40 indicating high stress. For this analysis, scores were dichotomized so that scores of 0–13 will be referred to as “low stress” and scores of 14–40 will be referred to as “high stress”.

#### Depression

Depression was measured by the Patient Health Questionnaire-8 (PHQ-8) [54]. We used a Spanish version of the PHQ-8 that has been validated [55] and tested for reliability [56] in the Spanish language. The PHQ-8 is scored from 0 to 24, with a higher score indicating more depression symptoms. When analyzed categorically, depression was defined using the CDC’s diagnostic cut-point for the PHQ-8, as scores greater than or equal to 10 [57].

#### Social support

Social support was measured by Spanish versions of the Perceived Social Support-Family (PSS-Family) scale and the Perceived Social Support-Friends (PSS-Friends) scale [58], both of which have been previously validated in the Spanish language and in Latin American contexts [59]. The PSS-Family is scored from 0 to 16, and the PSS-Friends is scored from 0 to 12, with higher scores indicating a higher level of support. Support questionnaires were dichotomized into low support and high support based on the distribution of the data.

#### Salivary cortisol

Salimetrics guides were used for maternal and infant saliva collection and storage protocols [60]. Maternal saliva samples were collected at 34–36 weeks of pregnancy and at one month postpartum. On both of these occasions, women provided three samples: one immediately upon waking (sample one), one 30 minutes after waking (sample two), and one prior to sleep (sample three). Women stored their samples in their freezers until they were collected by the study team the following day. Saliva samples were then stored at -20° C until analysis. Infant salivary samples were collected when the infant was three days old and two months old. At three days old, basal cortisol samples were collected to capture variation due to the prenatal environment with little postnatal influence. At two months of age, infant basal cortisol and cortisol reactivity were measured. Cortisol reactivity was measured as the difference between salivary cortisol levels before and 20–25 minutes after a stressor per a previously published infant stress reactivity protocol [61]. All infant saliva samples were collected using Salimetrics Infant Swabs and placed into Salimetrics Swab Storage tubes and frozen at -20° C until analysis.

#### HPA axis dysregulation

Maternal HPA axis dysregulation was assessed through morning cortisol and through cortisol awakening response (CAR), the difference between the cortisol concentrations of sample two and sample one. In our analyses, high morning cortisol and a blunted (low) CAR were considered to be measures of maternal HPA axis dysregulation. Cortisol dysregulation in infants was measured through elevated basal cortisol at three days old and two months old and a blunted or exaggerated cortisol reactivity at two months old. High basal cortisol [62] and blunted [61] and exaggerated [63] cortisol reactivity have been cited as evidence of infant cortisol dysregulation. Since there are no validated ways to define elevated cortisol in infants at this age, we dichotomized the sample into low and high cortisol concentrations at the median due to the small sample size.

#### Covariates

In addition to the scales, saliva samples, and stool samples, we used a sociodemographic survey at baseline that inquired about general sociodemographics, household size and composition, employment, parity, health behaviors, education, geographic history, and other themes. Data on obstetric and infant characteristics were collected at postpartum visits.

### Laboratory analysis

#### DNA isolation

Samples were transferred to a 2 mL tube containing 200 mg of ≤106 μm glass beads (Sigma, St. Louis, MO) and 0.3 mL of Qiagen ATL buffer (Valencia, CA), supplemented with 20 mg/mL lysozyme (Thermo Fisher Scientific, Grand Island, NY). The suspension was incubated at 37°C for 1 h with occasional agitation. Subsequently the suspension was supplemented with 600IU of Qiagen proteinase K and incubated at 60°C for 1 h. Finally, 0.3 mL of Qiagen AL buffer was added and a final incubation at 70°C for 10 minutes was carried out. Bead beating was then employed for 3 minutes in a Qiagen TissueLyser II at 30Hz. After a brief centrifugation, supernatants were aspirated and transferred to a new tube containing 0.3 mL of ethanol. DNA was purified using a standard on-column purification method with Qiagen buffers AW1 and AW2 as washing agents and eluted in 10mM Tris (pH 8.0).

#### 16S rRNA amplicon sequencing

12.5 ng of total DNA were amplified using universal primers targeting the V4 region of the bacterial 16S rRNA gene [64,65]. Primer sequences contained overhang adapters appended to the 5’ end of each primer for compatibility with Illumina sequencing platform. Master mixes contained 12.5 ng of total DNA, 0.2 μM of each primer and 2x KAPA HiFi HotStart ReadyMix (KAPA Biosystems, Wilmington, MA). Each 16S rRNA amplicon was purified using the AMPure XP reagent (Beckman Coulter, Indianapolis, IN). In the next step each sample was amplified using a limited cycle PCR program, adding Illumina sequencing adapters and dual‐index barcodes (index 1(i7) and index 2(i5)) (Illumina, San Diego, CA) to the amplicon target. The final libraries were again purified using the AMPure XP reagent (Beckman Coulter), quantified and normalized prior to pooling. The DNA library pool was then denatured with NaOH, diluted with hybridization buffer and heat denatured before loading on the MiSeq reagent cartridge (Illumina) and on the MiSeq instrument (Illumina). Automated cluster generation and paired–end sequencing with dual reads were performed according to the manufacturer’s instructions.

#### Salivary cortisol

Saliva samples were thawed and assayed in duplicate for salivary cortisol using commercially available enzyme-linked immunosorbent assay (ELISA) kits (Salimetrics, State College, PA) according to Salimetrics protocol [66]. Cortisol concentrations were log-transformed prior to analysis.

### Bioinformatics and statistical analysis

Sequencing output from the Illumina MiSeq platform were converted to fastq format and demultiplexed using Illumina Bcl2Fastq 2.18.0.12. The resulting paired-end reads were processed using QIIME 2 2018.11 [67]. Index and linker primer sequences were trimmed using the QIIME 2 invocation of cutadapt. The resulting paired-end reads were processed with DADA2 through QIIME 2 including merging paired ends, quality filtering, error correction, and chimera detection. Amplicon sequencing units from DADA2 were assigned taxonomic identifiers with respect to Green Genes release 13_08 using the QIIME 2 q2-feature-classifier. Alpha diversity (Faith PD whole tree, Evenness Shannon indexes), and observed species were estimated using QIIME 2 at a rarefaction depth of 5,000 sequences per sample. Beta diversity estimates were calculated within QIIME 2 using weighted and unweighted Unifrac distances as well as Bray-Curtis dissimilarity between samples at a subsampling depth of 5,000 reads/sample. Results were summarized, visualized through principal coordinate analysis, and significance was estimated as implemented in QIIME 2. Significance of differential abundance was estimated using ANCOM as implemented in QIIME 2.

Mann-Whitney U-tests were used to assess differences in alpha diversity (Shannon index values) based on precarity and cortisol measures. Principal Coordinates Analysis (PCoA) of weighted UniFrac distances matrices was used to determine clustering between two groups (eg. not depressed vs. depressed). Next, to examine the taxa distribution for the entire sample, we calculated relative abundances of bacteria within each taxon. Taxa that constituted less than 1% average relative abundance of the samples were excluded. Next, we conducted taxon-specific analysis to test for relationships between maternal precarity exposures and patterns of colonization using non-parametric Mann-Whitney U-tests, since relative abundances of individual taxa are not independent nor normally distributed. We also ran this analysis with False discovery rate (FDR) corrected p-values. The same was done for relationships between maternal cortisol and patterns of colonization as well as infant cortisol and patterns of colonization.

Finally, we ran adjusted linear regression models with robust standard errors to assess the effects of precarity and cortisol measures on phylum, family, and genus groups. In these models, we controlled for known confounders including mode of delivery, infant feeding [68–70], and also pre- or postpartum exposure to the exposure of interest in the model. For example, the model assessing the effects of friend support during pregnancy on relative abundance of taxa was adjusted for mode of delivery, infant feeding, and postpartum friend support, and the model assessing the effects of postpartum depression on relative abundance of taxa was adjusted for mode of delivery, infant feeding, and depression during pregnancy, etc. Infant feeding was a dichotomous variable defined by either exclusive breastfeeding or ever having been fed formula at two months of age. While some mothers reported having fed formula to their infants at least once, both cultural norms and the prohibitively high cost of formula in this setting made formula feeding rare. All infants who were formula-fed at some point were predominantly breastfed. No infant in the study was exclusively formula-fed and no infant had begun complementary feeding. These covariates were selected based on their persistent association with gut microbiome differentiation in the literature [68–70]. All regression analyses were conducted using Stata version Stata/MP 16.0 (StataCorp, College Station, TX).

## Results

### Precarity and cortisol descriptive data

In this sample, 12% of women were food insecure (Table 1). The majority of women experienced high stress during pregnancy (60%), but the experience of high stress decreased over time in the postpartum, so that by two months postpartum only 36% of women reported experiencing high stress. The prevalence of depression peaked at one month postpartum (29.2%) and decreased to only 16% by two months postpartum. While the levels of both low family and low friend support were least common in during pregnancy, each at 36%, low family support was most common at one month postpartum (46%), and low friend support was most common at two months postpartum (48%). Over half (56%) of the infants in the sample were born by Caesarean section, and 36% had received formula by two months postpartum. Correlations among precarity measures are shown in S1 Table.

**Table 1:** Maternal and infant characteristics.

**Maternal Characteristics**	**Mean (SD) or no. (%)**		
Age (years)		27.9 (5.9)	
Parity		0.96 (0.89)	
Married		22 (88%)	
Education			
Less than high school		3 (12%)	
Completed high school		16 (64%)	
Completed college		6 (24%)	
Born on Galápagos		9 (36%)	
**Obstetric Data**			
Gestational age (weeks)		38.3 (1.4)	
Cesarean delivery		14 (56%)	
**Infant Characteristics**			
Male offspring		15 (60%)	
Infant birth weight (g)		3346.4 (370.7)	
Formula feeding ^a^		9 (36%)	
**Precarity Measures**	**Pre-partum**	**Postpartum, 1 month**	**Postpartum, 2 months**
Food Insecure	12.0%	--	--
High stress	60.0%	50.0%	36.0%
High depression	24.0%	29.2%	16.0%
Low family support	36.0%	46.0%	40.0%
Low friend support	36.0%	38.0%	48.0%

^a^ Measures taken when the infant was 2 months old.

Food insecurity is defined as scoring a 6–15 on the Latin American and Caribbean Food Security Scale (ELCSA). High stress is defined as scoring a 14–40 on the Perceived Stress Scale (PSS). High depression is defined as scoring a 10 or higher on the Patient Health Questionnaire (PHQ-8). Family and friend support were dichotomized according to the distribution of the data, so that low family support is defined as scoring a 14 or lower on the Perceived Social Support-Family (PSS-Family) scale, and low friend support is defined as scoring a 9 or lower on Perceived Social Support-Friends (PSS-Friends) scale.

### Gut microbiome alpha diversity

**Fig 2:**
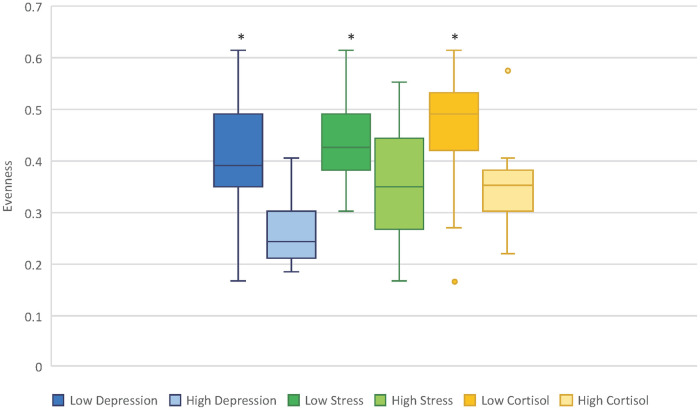
High postpartum maternal depression, stress, and morning cortisol concentration significantly decrease Shannon’s Entropy in infant stool. * Indicates *p* ≤ 0.05.

In the postpartum, maternal depression (*p* = 0.04), stress (*p* = 0.01), and high morning cortisol (*p* = 0.04) were all significantly associated with lower Shannon diversity values in infant stool at two months of age (Fig 2). Pre-partum measures of depression, stress, and maternal cortisol were not associated with differences in alpha diversity in infant stool at two months of age. Social support and food security in the peripartum period were also not associated with differences in alpha diversity in infant stool. Variations in Shannon diversity were not associated with either mode of delivery or infant feeding at *p* ≤ 0.05.

### Gut microbiome beta diversity and PCoA plots

**Fig 3:**
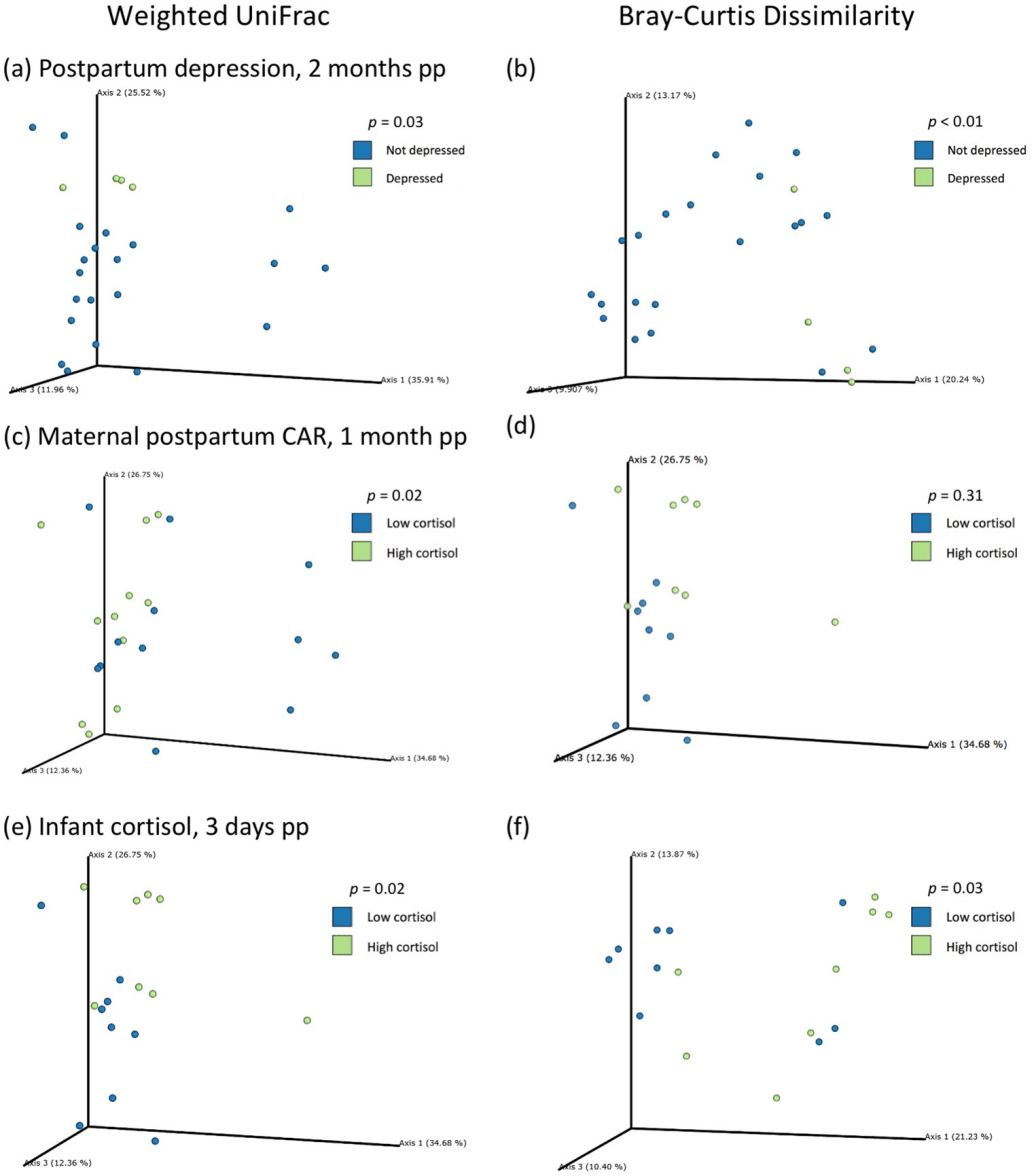
Stool sample sequences from infants cluster separately by maternal postpartum depression, maternal postpartum cortisol awakening response, and infant cortisol on a Principal Coordinates Analysis (PCoA) using weighted UniFrac distances and Bray-Curtis dissimilarity.

When weighted UniFrac distances and Bray-Curtis dissimilarities were plotted on PCoA plots, samples from infants whose mothers were depressed at two months postpartum clustered separately from samples of infants whose mothers were not depressed at two months postpartum (Fig 3). These differences were statistically significant for both weighted UniFrac distance (*p* = 0.03) and Bray-Curtis dissimilarity (*p* < 0.01). Measures of beta diversity in infant stool were not significantly different for any other precarity measures or for maternal depression experienced during pregnancy. Analyses of differences in beta diversity for maternal cortisol measures revealed that samples from infants whose mothers had a low postpartum CAR plotted separately from samples of infants whose mothers had a high CAR on PCoA plots of weighted UniFrac distances (*p* = 0.02), but not of Bray-Curtis dissimilarity (*p* = 0.31) (Fig 3). No other measures of maternal cortisol were associated with significantly different beta diversity in infant stool samples taken at two months of age. Last, when we tested differences in beta diversity of the infant gut microbiome based on infant cortisol, only basal infant cortisol at three days old was significantly associated with differences for weighted UniFrac distance (*p* = 0.02) and Bray-Curtis dissimilarity (*p* = 0.03) (Fig 3).

### Relative abundance of predominant taxa

Four phyla, Firmicutes, Actinobacteria, Proteobacteria, and Bacteroidetes accounted for 99.9% of the average total composition of infant gut microbiota (Table 2). Thirteen families and 13 genera, each of whose average relative abundance was greater than or equal to 1%, accounted for 95.7% and 92.3% of the average total composition of infant gut microbiota within each taxon, respectively.

**Table 2:** Average infant gut microbiota composition at the phylum, family, and genus taxa.

Phylum	% abundance	*Family*	% abundance	*Genus*	% abundance
Firmicutes	32.1%	*Erysipelotrichaceae*	6.0%	*Clostridium*	5.8%
		*Clostridiaceae*	5.2%	*Clostridium*	5.2%
		*Lachnospiraceae*	4.4%	*Clostridium*	2.2%
		*Peptostreptococcaceae*	4.3%	Unspecified	4.2%
		*Streptococcaceae*	4.0%	*Streptococcus*	3.7%
		*Lactobacillaceae*	2.9%	*Lactobacillus*	2.9%
		*Enterococcaceae*	2.4%	*Enterococcus*	2.3%
		*Veillonellaceae*	1.1%	*Veillonella*	1.0%
		*Staphylococcaceae*	1.0%	*Staphylococcus*	1.0%
Actinobacteria	30.8%	*Bifidobacteriaceae*	27.8%	*Bifidobacterium*	27.7%
		*Coriobacteriaceae*	2.0%	*Collinsella*	1.8%
Proteobacteria	23.4%	*Enterobacteriaceae*	23.0%	Unspecified	23.0%
Bacteroidetes	13.6%	*Bacteroidaceae*	11.6%	*Bacteroides*	11.6%
Other	0.1%	*Other*	4.3%	Other	7.7%

**Fig 4:**
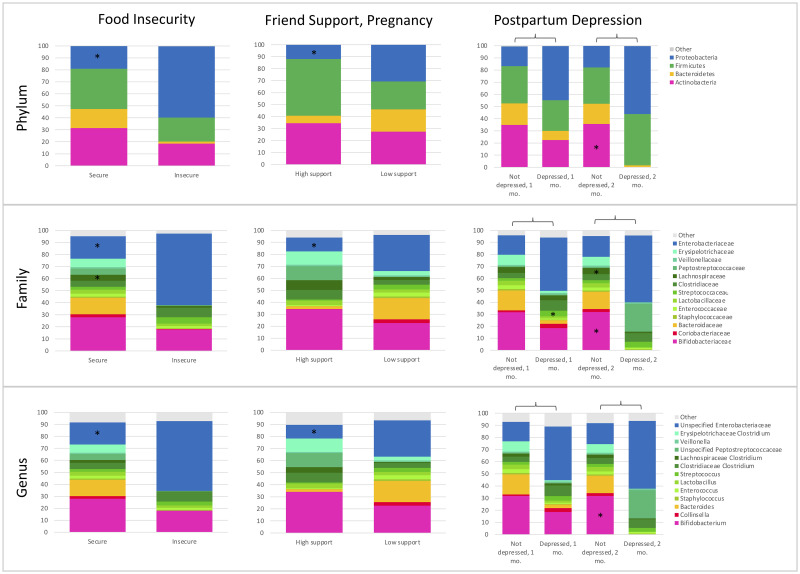
Food security, social support, and postpartum depression contribute to differential relative abundance of the phylum, family, and genus taxonomic levels in infant stool. * Indicates *p* ≤ 0.05.

**Fig 5:**
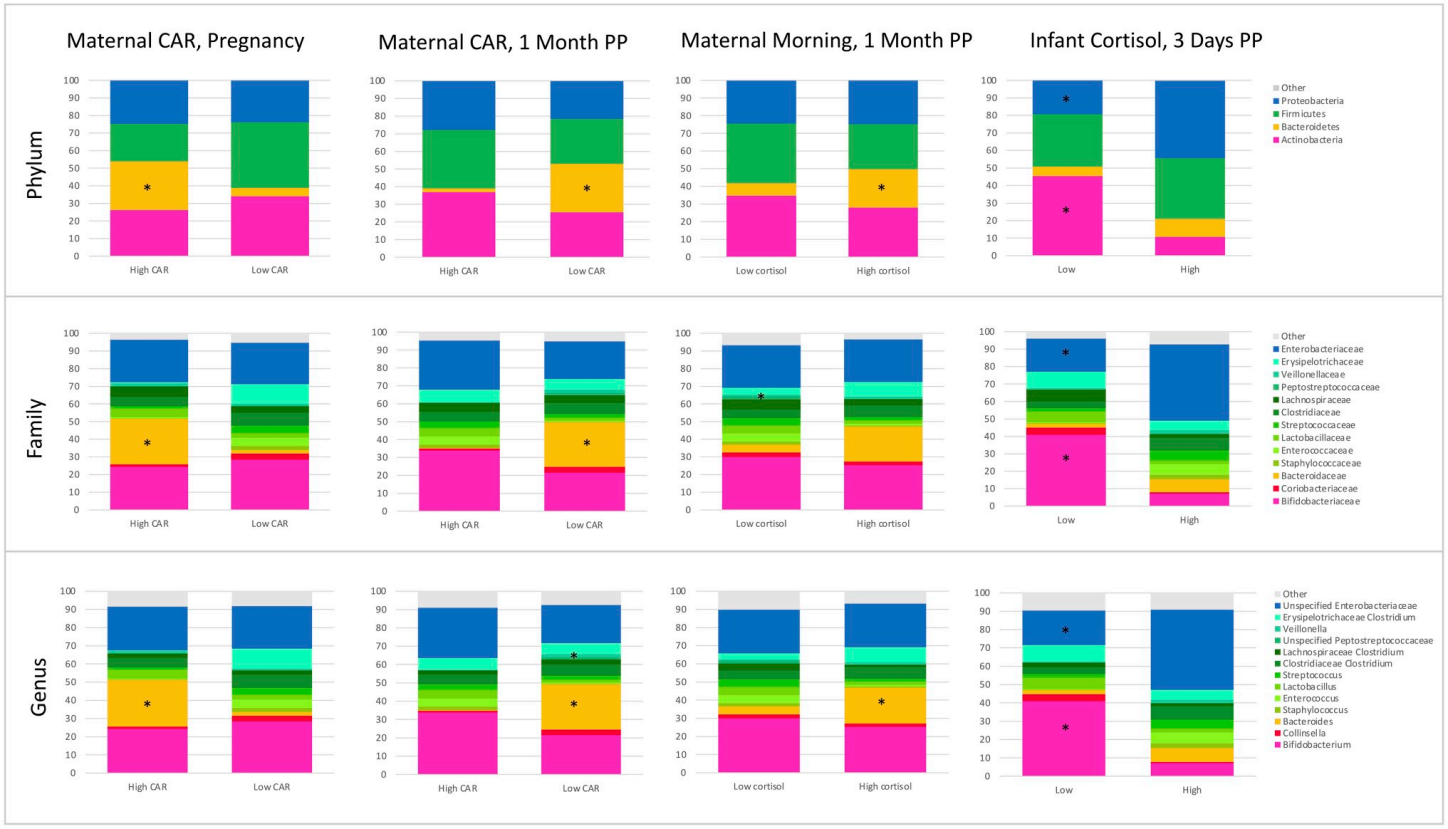
Maternal and infant cortisol are associated with differential relative abundance of the phylum, family, and genus taxonomic levels in infant stool. * Indicates *p* ≤ 0.05.

Mann-Whitney U tests and adjusted regression models were run for precarity and cortisol exposures with each of these phyla, families, and genera. Mann-Whitney U tests that were significantly associated (*p* ≤ 0.05) with differences in abundance are shown in Table 3, and associations that remain significant after applying the FDR correction are indicated in gray cells. Adjusted regression models that were significantly associated with differences in taxa abundance are shown in Table 4. Only significant associations are shown in Tables 3 and 4. Within the adjusted models, mode of delivery was significantly associated with differences in taxa abundance in approximately 50% of the models, while infant feeding was significantly associated with taxa abundance in <10% of models. Relative abundance plots for maternal precarity measures that were significant across taxa in adjusted models are shown in Fig 4, and relative abundance plots for maternal and infant cortisol measures that were significant across taxa in adjusted models are shown in Fig 5. Results of ANCOM analysis were consistent with these findings.

**Table 3:** Maternal precarity and maternal and infant HPA axis dysregulation are associated with differences in taxa abundance in bivariate analyses.

Exposure	Outcome	Median (low precarity)	Median (high precarity)	Z-value	p-value
**Maternal Precarity**					
*Phylum*					
Food Insecurity, pregnancy	Proteobacteria	16.00	59.11	-2.26	0.02
Family support, 1 month pp^a^	Proteobacteria	35.15	16.9	-1.94	0.05
Depression, 1 months pp	Proteobacteria	15.10	41.46	-2.00	0.04
Depression, 2 months pp	Actinobacteria	29.45	0.27	2.60	0.01
*Family*					
Food Insecurity, pregnancy	*Enterobacteriaceae*	15.06	58.99	-2.26	0.02
Family support, pregnancy	*Coriobacteriaceae*	0	0.41	2.11	0.04
Family support, 2 months pp	*Enterococcaceae*	0.25	0.15	-0.17	0.04
Friend support, pregnancy	*Streptococcaceae*	0.95	2.03	2.38	0.02
Friend support, pregnancy	*Lachnospiraceae*	9.65	0.14	-1.96	0.05
Friend support, 1 month pp	*Coriobacteriaceae*	0	0.55	2.42	0.02
Stress, pregnancy	*Clostridiaceae*	4.90	0.14	2.25	0.02
Stress, 2 months pp	*Clostridiaceae*	4.45	0.02	2.86	<0.01
Depression, 1 month pp	*Streptococcaceae*	1.26	5.18	-2.70	0.01
Depression, 1 month pp	*Enterobacteriaceae*	13.87	41.43	-2.00	0.05
Depression, 2 months pp	*Bifidobacteriaceae*	28.00	0.04	2.45	0.01
*Genus*					
Food Insecurity, pregnancy	*Unspecified Enterobacteriaceae*	15.06	59.00	-2.26	0.02
Family support, pregnancy	*Enterococcus*	0.61	0.06	-2.25	0.02
Friend support, pregnancy	*Streptococcus*	0.95	1.77	2.04	0.04
Friend support, pregnancy	*Lachnospiraceae Clostridium*	1.46	0	-2.14	0.04
Friend support, pregnancy	Unspecified *Peptostreptococcaceae*	0.13	0	-2.43	0.02
Friend support, pregnancy	*Collinsella*	0	0	2.36	0.03
Friend support, 1 month pp	*Collinsella*	0	0	2.34	0.04
Friend support, 2 months pp	*Collinsella*	0	0	2.10	0.04
Stress, pregnancy	*Clostridiaceae Clostridium*	4.90	0.14	2.25	0.02
Stress, 2 months pp	*Clostridiaceae Clostridium*	4.45	0.02	2.86	<0.01
Depression, 1 month pp	Unspecified *Enterobacteriaceae*	13.87	41.43	-2.00	0.05
Depression, 2 months pp	*Bifidobacterium*	27.99	0.04	2.45	0.01
**Maternal HPA Axis Dysregulation**					
*Phylum*					
Maternal CAR,1 month pp	Bacteroidetes	18.53	0.09	2.40	0.02
*Family*					
Maternal CAR, pregnancy	*Streptococcaceae*	2.17	0.84	2.39	0.02
Maternal CAR, 1 month pp	*Veillonellaceae*	1.17	0.17	2.06	0.04
Maternal morning, pregnancy	*Erysipelotrichaceae*	0.01	0.43	-2.46	0.01
Maternal morning, 1 month pp	*Peptostreptococcaceae*	0.12	0	1.94	0.05
*Genus*					
Maternal CAR, pregnancy	*Staphylococcus*	0.53	0.16	2.07	0.04
Maternal morning, pregnancy	*Erysipelotrichaceae Clostridium*	0	0.43	-1.99	0.05
**Infant HPA Axis Dysregulation**					
*Phylum*					
Infant basal, 3 days	Actinobacteria	42.42	8.98	2.50	0.01
*Family*					
Infant basal, 3 days	*Bifidobacteriaceae*	40.61	0.04	2.66	0.01
Infant basal, 3 days	*Streptococcaceae*	1.55	4.00	-2.12	0.03
*Genus*					
Infant basal, 3 days	*Bifidobacterium*	40.61	0.04	2.66	<0.01
Infant basal, 3 days	*Staphylococcus*	0.14	0.75	-2.12	0.04
Infant cortisol response, 2 months	*Staphylococcus*	0.45	0.12	2.02	0.05
Infant cortisol response, 2 months	*Streptococcus*	1.94	0.86	1.96	0.05
Infant cortisol response, 2 months	*Unspecified Peptostreptococcaceae*	0	0.07	-1.95	0.05

Table shows all significant results for bivariate analyses of precarity measures and relative abundance of phylum, family, and genus taxa at p ≤ 0.05

Gray cells are significant post-FDR correction.

^a^pp indicates postpartum.

**Table 4:** Maternal precarity and maternal and infant HPA axis dysregulation are associated with differences in taxa abundance in adjusted models.

Exposure	Outcome	Model R^2^	Variable β^1^ (SE)	Standardized β (95% CI)	p-value
**Maternal Precarity**					
*Phylum*					
↑ Food Insecurity, pregnancy	↑ Proteobacteria	0.37	33.67 (15.89)	0.43 (0.01, 0.84)	0.05
↓ Friend support, pregnancy	↑ Proteobacteria	0.46	-29.02 (9.13)	-0.59 (-0.98, -0.20)	0.01
↑ Depression, 2 months pp^a^	↓ Actinobacteria	0.24	-31.20 (14.26)	-0.41 (-0.80, -0.02)	0.04
*Family*					
↑ Food Insecurity, pregnancy	↑ *Enterobacteriaceae*	0.37	33.63 (16.02)	0.42 (0.00, 0.85)	0.05
↑ Food Insecurity, pregnancy	↓ *Lachnospiraceae*	0.37	-6.63 (2.23)	-0.33 (-0.56, -0.10)	0.01
↓ Friend support, pregnancy	↑ *Enterobacteriaceae*	0.47	-28.97 (9.02)	-0.59 (-0.97, -0.21)	<0.01
↑ Depression, 1 month pp	↑ *Streptococcaceae*	0.13	3.53 (1.16)	0.38 (0.12, 0.65)	0.01
↑ Depression, 2 months pp	↓ *Bifidobacteriaceae*	0.21	-29.16 (12.99)	-0.40 (-0.77, -0.03)	0.04
↑ Depression, 2 months pp	↓ *Lachnospiraceae*	0.47	-7.21 (2.38)	-0.43 (-0.73, -0.14)	0.01
*Genus*					
↑ Food Insecurity, pregnancy	↑ unspecified *Enterobacteriaceae*	0.37	33.63 (16.06)	0.42 (0.00, 0.85)	0.05
↓ Family support, pregnancy	↑ *Bifidobacterium*	0.41	-38.48 (11.21)	-0.70 (-1.13, -0.28)	<0.01
↓ Family support, 2 months pp	↓ *Bifidobacterium*	0.41	36.63 (11.13)	0.67 (0.25, 1.09)	<0.01
↓ Friend support, pregnancy	↑ unspecified *Enterobacteriaceae*	0.47	-29.13 (9.01)	-0.59 (-0.97, -0.21)	<0.01
↑ Depression, 2 months pp	↓ *Bifidobacterium*	0.21	-29.06 (12.95)	-0.40 (-0.77, -0.03)	0.04
**Maternal HPA Axis Dysregulation**					
*Phylum*					
↓ Maternal CAR, pregnancy	↓ Bacteroidetes	0.61	22.32 (9.27)	0.47 (0.05, 0.89)	0.03
↓ Maternal CAR, 1 month pp	↑ Bacteroidetes	0.75	-23.05 (6.42)	-0.49 (-0.78, -0.20)	<0.01
↑ Maternal morning, 1 month pp	↑ Bacteroidetes	0.33	24.76 (9.98)	0.53 (0.07, 0.98)	0.03
*Family*					
↓ Maternal CAR, pregnancy	↓ *Bacteroidaceae*	0.63	24.65 (8.46)	0.51 (0.14, 0.89)	0.01
↓ Maternal CAR, 1 month pp	↑ *Bacteroidaceae*	0.76	-21.37 (6.00)	-0.45 (-0.71, -0.18)	<0.01
↓ Maternal CAR, 1 month pp	↑ *Veillonellaceae*	0.44	-1.39 (0.49)	-0.41 (-0.71, -0.10)	0.01
*Genus*					
↓ Maternal CAR, pregnancy	↓ *Bacteroides*	0.63	24.65 (8.46)	0.51 (0.14, 0.89)	0.01
↓ Maternal CAR, 1 month pp	↑ *Bacteroides*	0.76	-21.37 (6.00)	-0.45 (-0.71, -0.18)	<0.01
↓ Maternal CAR, 1 month pp	↑ *Veillonella*	0.31	-1.41 (0.48)	-0.42 (-0.73, -0.11)	0.01
↑ Maternal morning, 1 month pp	↑ *Bacteroides*	0.57	27.79 (9.55)	0.58 (0.16, 1.00)	0.01
**Infant HPA Axis Dysregulation**					
*Phylum*					
↑ Infant basal, 3 days	↓ Actinobacteria	0.65	-35.69 (8.13)	-0.64 (-0.96, -0.32)	<0.01
↑ Infant basal, 3 days	↑ Proteobacteria	0.48	26.29 (11.00)	0.55 (0.05, 1.05)	0.03
*Family*					
↑ Infant basal, 3 days	↓ *Bifidobacteriaceae*	0.60	-34.65 (8.02)	-0.65 (-0.98, -0.32)	<0.01
↑ Infant basal, 3 days	↑ *Enterobacteriaceae*	0.48	26.15 (11.03)	0.55 (0.04, 1.05)	0.04
*Genus*					
↑ Infant basal, 3 days	↓ *Bifidobacterium*	0.60	-34.65 (8.01)	-0.65 (-0.98, -0.32)	<0.01
↑ Infant basal, 3 days	↑ unspecified *Enterobacteriaceae*	0.48	26.11 (11.06)	0.55 (0.04, 1.05)	0.03

Table shows all significant models for precarity measures and relative abundance of phylum, family, and genus taxa at p ≤ 0.05.

All models control for mode of delivery and infant feeding. Pre-partum models control for postpartum precarity measures. Postpartum models control for pre-partum precarity measures.

^a^pp indicates postpartum.

^1^ indicates unstandardized.

Of the measures of maternal precarity, food insecurity, low friend support, low family support, and postpartum depression were significantly associated with differences in taxa abundance in infant stool in controlled models run with robust standard errors (Table 4). Food insecurity was associated with a higher relative abundance of Proteobacteria (*p* = 0.05), specifically an unspecified genus of *Enterobacteriaceae* (*p* = 0.05), as well as a lower relative abundance of the family *Lachnospiraceae* (*p* = 0.01) (Table 4). Low friend support during pregnancy was associated with a higher relative abundance of Proteobacteria (*p* = 0.01), specifically an unspecified genus of *Enterobacteriaceae* (*p* < 0.01). Postpartum depression was associated with a lower relative abundance of Actinobacteria (*p* = 0.04), specifically the genus *Bifidobacterium* (*p* = 0.04). It was also associated with a lower abundance of the family *Lachnospiraceae* (*p* = 0.01) and a higher relative abundance of the family *Streptococcaceae* (*p* = 0.01). Notably, low family support during pregnancy was associated with higher relative abundance of the genus *Bifidobacterium* (*p* < 0.01), while low family support in the postpartum was associated with a lower abundance of bifidobacteria (*p* < 0.01). Stress was not associated with differences in taxa.

In adjusted models, maternal HPA axis dysregulation measures were associated with differences in taxa both during and after pregnancy (Table 4). Notably, maternal CAR during and after pregnancy were each associated with differences in *Bacteroidetes*, and particularly the genus *Bacteroides*, but in different directions. A low maternal CAR during pregnancy was associated with a lower abundance of *Bacteroidetes* (*p* = 0.03) and *Bacteroides* (*p* = 0.01), and a low maternal CAR in the postpartum was associated with a higher abundance of these taxa (*p* < 0.01 for each) as well as a higher abundance of *Veillonella* (*p* = 0.01). Maternal morning cortisol during pregnancy was not associated with differences in infant gut taxa, but high postpartum morning cortisol was associated with a greater relative abundance of *Bacteroidetes* (*p* = 0.03), and in particular the genus *Bacteroides* (*p* = 0.01).

Infant cortisol concentrations at three days postpartum, but not two months postpartum, were associated with differences in infant gut taxa in adjusted models (Table 4). High infant basal cortisol at three days postpartum was associated with a lower abundance of Actinobacteria (*p* < 0.01), and specifically the genus *Bifidobacterium* (*p* < 0.01), and a higher abundance of Proteobacteria (*p* = 0.03), particularly an unspecified genus of *Enterobacteriaceae* (*p* = 0.03) (Table 4).

## Discussion

In this study, we examined how multiple facets of women’s precarity and HPA axis functioning in the peripartum period contribute to the development of their infants’ gut microbiome communities and associated HPA axis functioning. We found support for all three of our proposed pathways, suggesting that: 1) maternal peripartum precarity is associated with differences in infant gut microbiota diversity and taxa abundance, 2) peripartum maternal HPA axis functioning is associated with the infant gut microbiome, and 3) differences in relative abundance of taxa in the infant gut are associated with differences in infant HPA axis functioning.

Our results show support for our first proposed pathway, that maternal precarity during and after pregnancy is associated with differences in infant gut microbiota diversity and predominant taxa. Overall, women’s experiences of precarity in the Galápagos during the peripartum period were associated less diverse, more potentially pathogenic, and less protective bacteria in the infant gut.

Prenatal exposures to precarity, including food insecurity and low social support, were associated with a higher relative abundance of Proteobacteria, a phylum that contains many pathogenic and opportunistic bacteria [24], in infant stool. Within the Proteobacteria phylum, these infants had a higher relative abundance of an unspecified genus of *Enterobacteriaceae*, a family of bacteria that includes the pathogenic genera *Escherichia* (including *Escherichia coli*), *Enterobacter*, and *Salmonella* [24]. Further, *Enterobacteriaceae* are known to produce lipopolysaccharides (LPS), which stimulate the HPA axis [71] and have been associated with inflammation in a variety of metabolic diseases [72]. A high relative abundance of *Enterobacteriaceae* in early infancy has been associated with risk for allergy and eczema [73]. Nonetheless, not all Proteobacteria nor *Enterobacteriaceae* are pathogenic, and more specificity is needed in future research to discern pathways to long-term health. Prenatal precarity was also associated with a lower abundance of *Lachnospiraceae*, a family of bacteria that promotes gut health and has been shown to be protective against obesity and insulin resistance in mice [74] and decrease risk for heart failure in humans [75]. Further, we found that low family social support during pregnancy is associated with a higher relative abundance of bifidobacteria in infants. In contrast to other studies that have examined this pathway and in contrast to our hypothesis, we did not find that offspring exposed prenatally to maternal precarity have lower abundances of lactobacilli and bifidobacteria. One study found these associations in humans [24], while others have found similar results in animal models [22], where offspring of monkeys stressed during pregnancy had significantly lower abundance of bifidobacteria and lactobacilli at two days after birth [49], and offspring of mice stressed during pregnancy had a significantly lower abundance of *Lactobacillus* [16]. It is possible that we did not observe this finding due to the potential buffering effect of breastfeeding.

Nonetheless, and in contrast to our prenatal precarity exposures, we observed that various postpartum maternal precarity measures were associated with a lower relative abundance *Bifidobacterium*, as well as a lower abundance of *Lachnospiraceae* and *Streptococcaceae* in infant stool. Our finding that maternal precarity is associated with a lower abundance of *Bifidobacterium* is of particular interest since most bifidobacteria are anaerobic, anti-inflammatory bacteria known to be one of the cornerstones of a healthy infant gut microbiome [76]. A high abundance of bifidobacteria has been associated with reduced risk for allergic disease [77,78] and excessive weight gain [79,80], and a low abundance has been associated with increased crying in infants [81]. The observed difference in bifidobacteria abundance in our study is consistent with other literature that has found that bifidobacteria is sensitive to environmental perturbations [24], including preterm birth [82], antibiotic exposure [83], and Caesarean section [84], which have all been associated with a lower abundance of *Bifidobacterium* in the infant gut. Further, in addition to a lower abundance of *Lachnospiraceae*, whose benefits are discussed above, postpartum precarity was associated with a higher relative abundance of *Streptococcaceae*, whose genus *Streptococcus* has been associated with higher waist circumference [85], cardiometabolic diseases [86], and inflammatory diseases [87]. In general, maternal peripartum precarity was associated with a lower abundance of protective bacteria including bifidobacteria, and a higher abundance of pathogenic bacteria, including *Enterobacteriaceae* in infant stool.

Our analyses demonstrate mixed results for our second pathway, which assessed whether peripartum maternal cortisol is associated with infant gut microbiota composition. Our results again demonstrate contradictory microbial colonization patterns between maternal cortisol dysregulation during pregnancy and in the postpartum. Here, a low maternal CAR (an indicator of HPA axis dysregulation) during pregnancy was associated with a lower abundance of *Bacteroidetes*, and specifically *Bacteroides*, while a low maternal CAR in the postpartum was associated with a higher abundance of *Bacteroidetes* and *Bacteroides*. Further, in the postpartum, high maternal morning cortisol was also associated with a higher relative abundance of *Bacteroidetes* and *Bacteroides*. This difference in colonization based on the timing of maternal HPA axis dysregulation could be due to the mechanism of colonization. Prenatal stress (measured through a low CAR) may have decreased *Bacteroidetes* in the mother’s own microbiota, which the infant was exposed to *in utero* and during birth, while postpartum maternal stress may have shaped infant biology through physical environmental exposures, breastfeeding, or parenting behaviors. Other work in the Galápagos and elsewhere has found that *Bacteroidaceae* [88] and *Bacteroides* [84] are higher in infants born vaginally and those who are formula-fed. Low maternal CAR in the postpartum was also associated with a higher abundance of *Veillonella*, which has been associated with more cardiometabolic risk factors in adults [85].

Last, our results demonstrate support for our third pathway, which analyzed whether infant gut microbiota composition was associated with differences in infant cortisol in the postpartum. Notably, our results showed that shifts in microbiota colonization associated with infant HPA axis dysregulation mirror major microbial shifts associated with maternal precarity, including a lower abundance of Actinobacteria, specifically the genus *Bifidobacterium*, and a higher abundance of Proteobacteria, specifically an unspecified genus in the family *Enterobacteriaceae*. Other research has also found that bifidobacteria are intricately involved with the HPA axis. One study found that the reconstitution of bifidobacteria was associated with a decrease in stress responses in mice [12]. Further, this association is of particular interest since prenatal exposure to maternal stress has often been associated with a lower abundance of bifidobacteria in the infant gut [24,49]. Together, this evidence, along with our own results, suggests that maternal stress may influence infant HPA axis activity through microbial agents, though research is limited.

Beyond differences in taxa abundances, we found that in the postpartum, two measures of maternal precarity (depression and stress) and one measure of maternal HPA axis dysregulation (high morning cortisol) were associated with significantly lower alpha diversity. Studies have found that in adults, high microbial diversity has been associated with relatively more anti-inflammatory bacteria, while low microbial diversity has been associated with relatively more pro-inflammatory bacteria as well as higher adiposity and inflammation [89], suggesting that these stress-related differences in microbial diversity may lay the foundation for unfavorable consequences in the long-term. Nonetheless, a meta-analysis found that in infants under six months of age, alpha diversity (Shannon index) was higher in non-exclusively breastfed infants, complicating our understanding of a healthy infant gut [90]. Notably, we did not observe differences in alpha diversity for any maternal precarity or HPA axis dysregulation exposure during pregnancy. Since alpha diversity continues to develop throughout infancy [69], this observation could be a consequence of postpartum exposures on infant gut microbiome development.

Despite the fact that we found support for some measures in each of our proposed pathways, other measures within each pathway were not significantly associated with differences in gut microbiome development, demonstrating the complexity of measuring stress. Notably, maternal “stress” itself, measured on the PSS, was not associated with any differences in taxa abundance in adjusted models either during or after pregnancy, though postpartum stress was associated with lower alpha diversity. Our results are in contrast to other work that has found that infants of mothers with high reported stress have a higher relative abundance of proteobacterial groups and a lower relative abundance bifidobacteria [24]. Our results may be due to the fact that we used the PSS to measure stress, while other studies have utilized measures of stress and anxiety to indicate stress. Nonetheless our analyses show similar results borne out through other measures of maternal precarity in this context, including maternal food insecurity, low social support, and depression.

Our results demonstrate the importance of using a contextually-specific and multi-faceted approach to interrogate these complex biological pathways. By using four measures of maternal precarity on the Galápagos, food insecurity, low social support, depression, and stress, we were able to examine women’s experiences more fully. Further, many differences in microbiota composition were consistent across measures of precarity, suggesting an internal consistency among measures. For example, both postpartum stress and depression were associated with lower alpha diversity, and various measures of precarity were associated with lower *Bifidobacterium* abundance and higher *Enterobacteriaceae* abundance even in adjusted models. Notably, this same pattern of colonization was observed in infants whose HPA axis was dysregulated, lending support for hypotheses regarding communication between of the brain and the gut and reinforcing the importance of examining these intertwined pathways with a variety of both psychosocial and biological measures. To our knowledge, this is only the second study to assess the effects of maternal prenatal stress on infant gut microbiome development and associated HPA axis function in humans, and it is the first study to include both prenatal and postnatal stress in these models. Despite the fact that postnatal environments are known to contribute to infant gut composition [69,88], many studies on early gut development focus only on prenatal stress exposures. A few studies with animal models have tested the effects of infant postnatal stress on gut microbiome development [2,25], but to our knowledge, none of have tested this relationship in humans, which may be due to methodological limitations of examining stress in infants. In the present study, we use both maternal precarity and HPA axis function measures that could affect infant stress as well as measures of infant HPA functioning to examine infant stress in the postnatal period. Our results add to the literature on the intergenerational transmission of stress through the microbiome in the peripartum period in humans.

Nonetheless, these results have several important limitations. First, our small sample size (n = 25) allows us to test associations between variables, but it limits our statistical power, so the results presented here should be considered exploratory. Despite this, we estimate that we enrolled over half of all births on San Cristóbal in 2018 based on annual birth rates, so we believe that these results are generalizable to the island’s population. Second, since there is no standard for dichotomizing infant basal cortisol, we defined high and low infant basal cortisol based on the median. This approach allowed for even sample sizes in each group and is an exploratory method, but other methods for dichotomizing infant cortisol should be tested in larger samples. Next, while pathways between infant gut microbiome composition and infant HPA axis functioning are evident, our analysis cannot distinguish directionality. Further, the pathways in our models would be elucidated more clearly with a measurement of maternal gut or vaginal microbiome composition, which we did not have in this study. Last, the limitations of two covariates, mode of delivery and infant feeding are important to consider. First, mode of delivery included the categories of vaginal birth or Caesarean section, but we did not have data for membrane rupture, which could limit our understanding of which bacteria infants exposed to during birth. Second, infant feeding was split into two categories, “exclusive breastfeeding” and “ever received formula.” While formula feeding does induce important differences to the infant gut microbiome [68], we did not have data on how often infants in the “ever formula” category were formula fed, and therefore we could not assess dose effects. Nonetheless, other work has suggested that infants who have ever received formula do have significant differences in their gut microbiome compositions than infants who are exclusively breastfed [90,91], suggesting that this distinction is important. Despite these limitations, significant and consistent differences in infant gut microbiota composition are associated with measures of maternal precarity, and this work lays a foundation for further testing of these pathways in human models.

Overall, we find that exposures to maternal precarity and HPA axis dysregulation are associated with an increase in potentially pathogenic bacteria, including *Enterobacteriaceae*, *Streptococcaceae*, *and Veillonella*, and a decrease in potentially protective bacteria, including *Bifidobacterium* and *Lachnospiraceae*, as well as a decrease in alpha diversity. This initial colonization may permanently alter the infant’s neurodevelopment through changes to the synthesis of neuroinflammatory cytokines, neuromodulators, neurotransmitters, and the HPA axis, leaving the individual more susceptible to neuropsychiatric disease later in life [16,92]. Further, unfavorable changes to an individual’s foundational gut microbiome may increase risk of metabolic disease, autoimmune disease and allergy, and intestinal inflammation [5,9,92]. Our results suggest that maternal precarity may be an important factor in early infant gut microbiome composition, and that patterns of infant gut colonization may cause or respond to differences in infant HPA axis development. Further research is needed to further elucidate the relationships among these important pathways.

## Supporting information

S1 Table Correlations among precarity measures, represented as correlation coefficients (*r*). (DOCX)

S1 File (CSV)
