# Supplementary material for: Maternal precarity and HPA axis functioning shape infant gut microbiota and HPA axis development in humans
Source: PLoS One. 2021 May 20;16(5):e0251782. doi: 10.1371/journal.pone.0251782 (PMC8136730; doi:10.1371/journal.pone.0251782)
Supplement: S1 Table — (DOCX) [file pone.0251782.s001.docx]

**S1 Table.** **Correlations among precarity measures, represented as correlation coefficients (*r*).**

| **Precarity Measures** |  | Stress | | | Depression | | | Family Support | | | Friend Support | | |
| --- | --- | --- | --- | --- | --- | --- | --- | --- | --- | --- | --- | --- | --- |
|  |  | V1^a^ | V3 | V4 | V1 | V3 | V4 | V1 | V3 | V4 | V1 | V3 | V4 |
| Food Insecurity | V1 | 0.25 | **0.56*** | **0.41*** | **0.40*** | 0.35 | **0.58*** | 0.13 | -0.05 | -0.11 | 0.14 | 0.08 | 0.06 |
| Stress | V1 |  | **0.47*** | **0.55*** | **0.43*** | 0.29 | 0.35 | -0.23 | -0.16 | -0.22 | -0.03 | 0.00 | -0.10 |
|  | V3 |  |  | **0.53*** | 0.36 | **0.73*** | **0.58*** | 0.03 | -0.07 | -0.16 | -0.09 | -0.22 | -0.21 |
|  | V4 |  |  |  | **0.65*** | **0.43*** | **0.63*** | -0.26 | -0.32 | **-0.41*** | -0.05 | -0.33 | -0.28 |
| Depression | V1 |  |  |  |  | **0.54*** | **0.63*** | -0.15 | -0.32 | **-0.38*** | -0.28 | -0.30 | -0.29 |
|  | V3 |  |  |  |  |  | **0.71*** | -0.10 | -0.14 | -0.25 | -0.30 | -0.31 | -0.31 |
|  | V4 |  |  |  |  |  |  | 0.10 | 0.05 | -0.06 | -0.07 | -0.17 | -0.20 |
| Family support | V1 |  |  |  |  |  |  |  | **0.82*** | **0.82*** | **0.43*** | **0.46*** | **0.52*** |
|  | V3 |  |  |  |  |  |  |  |  | **0.94*** | 0.21 | 0.36 | 0.35 |
|  | V4 |  |  |  |  |  |  |  |  |  | 0.15 | 0.33 | 0.36 |
| Friend support | V1 |  |  |  |  |  |  |  |  |  |  | **0.77*** | **0.80*** |
|  | V3 |  |  |  |  |  |  |  |  |  |  |  | **0.86*** |
|  | V4 |  |  |  |  |  |  |  |  |  |  |  |  |

* Indicates *p* ≤ 0.05

^a^V1, V3, and V4 refer to Visits 1, 3, and 4, and reflect data from the visits at 34-36 of weeks gestation, one month postpartum, and two months postpartum respectively
